# Does Birth Month Matter? Effects of Birth Subgroupings on Motor Performance and Ball-Involving Tests in a Youth Soccer Academy

**DOI:** 10.3390/sports13110382

**Published:** 2025-11-04

**Authors:** Lorenzo Marcelli, Fioretta Silvestri, Gianluca Di Pinto, Andrea Colombo, Federica Marzoli, Maria Chiara Gallotta, Laura Guidetti, Fabrizio Perroni, Davide Curzi

**Affiliations:** 1 Department of Economic, Psychological, Communication, Education and Movement Science, University Niccolò Cusano, 00166 Rome, Italy; 2 BIND-Behavioral Imaging and Neural Dynamics Center, University “G. d’Annunzio” Chieti-Pescara, 66100 Chieti, Italy; 3 Department of Medicine and Aging Sciences, University “G. d’Annunzio” Chieti-Pescara, 66100 Chieti, Italy; 4 Department of Physiology and Pharmacology “Vittorio Erspamer”, Sapienza University of Rome, 00185 Rome, Italy; 5 Department of Biomolecular Sciences, Section of Exercise and Health Sciences, Carlo Bo University of Urbino, 61029 Urbino, Italy

**Keywords:** football, relative age effect, young players, chronological age, talent identification, agility, CMJ, dribbling, technical skills, player development

## Abstract

Youth motor performance changes are not strictly linear during their sports career, and within-year birth timing may introduce relative age differences, known as the relative-age effect. In a cross-sectional study of 170 male young soccer players (8–12 years old), field tests were compared between adjacent age groups and within each of them: countermovement jump (with and without arm swing); 15 m linear sprint and agility test with and without the ball. Non-parametric tests with post hoc comparisons revealed significant differences between consecutive birth years in physical skills, particularly between the youngest and oldest groups. Specifically, differences were noted in the jumping test between U12 and U11, and between U10 and U9. Additionally, linear sprint and agility tests showed significance in U13 versus U12 and U10 versus U9. Finally, dribbling skills mature later (in both tests, U13 v U12, U12 v U11; with the ball, U10 v U9). Differences were found within the groups based on the semester, but not the trimester of birth, confirming a progressive yet non-linear pattern and semester-level within-year differences. These findings suggest the key role of motor skills development trajectories in creating individualized training programs tailored to the needs of individual young soccer players.

## 1. Introduction

Soccer is an open-skill team sport involving a combination of motor (e.g., strength, endurance and flexibility) and technical skills as key indicators of performance, particularly in young players [1,2]. Some physical characteristics, such as cardiovascular fitness, follow a direct improvement with age, while others follow a non-linear trend (e.g., vertical counter-movement jump, slalom agility) [3].

Fundamental motor skills, being the foundation of movement in children, play a crucial role in overall motor competence [4,5]. The object control skills, a subcategory of fundamental motor skills, involve the manipulation of objects with hands, feet, or tools, requiring high levels of coordination [4]. Its development at an early age is considered essential to facilitate skills transfer [4]. Indeed, studies have shown that the most successful players possess advanced motor abilities from an early stage and maintain their advantage over future non-elite players throughout their development [6]. Furthermore, object control skills are positively related to age [7], and specific physical exercise interventions have significantly improved object control skills in children [5]. The evidence regarding soccer-specific skills is unclear, with some authors [2] claiming a strong relationship between physical and technical skills, while others have not reported any direct correlation [8]. Indeed, Duncan and colleagues [8] suggest that the main predictors of performance development should be identified in the fundamental motor skills and perceived competence, regardless of the player’s chronological age. Research findings confirm that physical maturity is a determining factor in physical performance, particularly in strength and muscular power. Biologically more mature athletes often outperform their less mature peers, although this relationship may vary according to chronological age, sex and the specific fitness component analyzed [1,9,10].

Although differences in physical performance across age groups are more closely related to differences in physical maturity, within the same birth years, selection processes often show a bias toward athletes born in the first months of the year, a phenomenon known as the Relative Age Effect (RAE) [11,12,13]. The advantage of being born in the first months of the year has been attributed to biological maturation, but it is also present when the differences in performance were corrected by somatic maturation [14]. RAE is more evident in younger age groups and tends to decrease with growth [15]. It appears to be present across all aspects of life (school, sport, lifestyle, etc.). Despite that, youth competitions are traditionally organized into age groups based on a fixed cut-off date (i.e., 1 January), but there is a growing interest in grouping players based on biological rather than chronological age, to address developmental differences [16,17]. Even by aligning the cut-off date to the academic year (September to August), RAE remains present as it should be addressed as an intrinsic factor of athletes, being non-eliminable [13]. In addition to physical performance, RAE also influences cognitive development and reflects broader maturation processes [18]. Studies have shown that growth goes hand in hand with improvements in both physical and academic performance [1,2,8,9,16,19].

Although the scientific literature has investigated the different levels of motor skills among young soccer players born in adjacent years, through field-based motor tests [2,9], few studies have examined differences in subjects born in the same year. Therefore, the aims of this study were to compare motor and dribbling skills in young soccer players through field-based tests (1) across consecutive age groups and (2) within the same birth year through birth-based subgrouping strategies. In our hypothesis, relatively older players (born earlier in the selection year) would demonstrate superior motor (sprint, CMJ, agility, flexibility) and technical (speed dribbling) performance compared to later-born peers.

## 2. Materials and Methods

### 2.1. Participants

One hundred and seventy young male soccer players were included in this study and were firstly divided into the following groups, based on their age range: Under 9 (*n* = 23, age = 8.2 ± 0.3 years), Under 10 (*n* = 31, age = 9.4 ± 0.3 years), Under 11 (*n* = 39, age = 10.4 ± 0.3 years), Under 12 (*n* = 42, age = 11.3 ± 0.3 years) and Under 13 (*n* = 35, age = 12.3 ± 0.3 years). The division into traditional age groups (U9–U13) reflects the official categories used in Italian and European youth competitions (FIGC/UEFA). To investigate the relative age effect (RAE) within the same age category, each group was further divided into two subgroups based on the period of birth: the oldest athletes (O), born in the first half of the year (January–June, subgroup O) and the youngest athletes (Y), born in the second half of the year (July–December, subgroup Y). Despite the limited sample size, an explorative analysis was performed to check magnitudes and potential differences within a shorter birth timeframe. The trimestral subdivision was organized as follows: Q1: January–March; Q2: April–June; Q3: July–September; and Q4: October–December [20,21].

Participants were selected from an Italian amateur soccer club (Soccer Club Frascati, Rome, Italy), based on their regular participation in both training and official matches. The club is located in a suburban Rome area with a relatively homogeneous socioeconomic population. Position-specific analysis was not conducted as young players at these ages often rotate between positions and do not have fixed tactical roles. To be included in the study, athletes had to have been active for the entire competitive season and be free from chronic or acute injuries in the three months preceding the study and during the whole season. Additionally, all participants had to have played soccer for at least two years prior to the start of the study.

All tests were conducted in October, at the beginning of the competitive season, after one month of training from the start of the activity in September, ensuring that players had adapted to the initial training phase before the test. The participants were homogeneous regarding their training status (e.g., average training hours per week: 4.5, divided into three training sessions; total training weeks per season: 40), as none of the participants underwent any strenuous activity or training outside of their normal training schedule.

Athletes and their parents were informed about the project through a written document, and informed consent was obtained from the athletes’ parents or legal guardians, as all participants were minors. Ethical approval was granted by the Institutional Review Board of the University Niccolò Cusano, study protocol number MO 8/22, in accordance with the 1964 Helsinki Declaration and its later amendments or comparable ethical standards.

### 2.2. Testing Procedures

During the two weeks prior to the data collection, all participants attended four familiarization sessions in which each test was explained, demonstrated, and practiced. To assess the physical and technical abilities (i.e., dribbling skills) of the players, the following tests were conducted on a synthetic grass field under stable weather conditions (temperature 18–22 °C, minimal wind). All participants wore standard soccer boots and uniform sports clothing. The selected tests represent fundamental components of youth soccer performance and were chosen based on their relevance to soccer-specific skills and established validity in youth populations. Tests were administered in a fixed order (warm-up; flexibility; CMJ; sprint; agility) with 3 min of recovery between different test types to minimize fatigue effects. On test day, participants were allowed an additional warm-up attempt before official recording.

Test:

- Counter Movement Jump (CMJ) and Counter Movement Jump with Arm Swing (CMJA). The players performed three consecutive vertical jumps from a standing position, which were measured using the Optojump System (96 LEDs, 1.0416 cm resolution, Microgate, Bolzano, Italy). The system allowed precise measurement of the jump height by analyzing the flight time of each jump [22]. The highest jump among the three attempts was recorded for analysis. Arm movements were standardized: in CMJ, arms remained positioned on the hips throughout the movement, while in CMJA, free arm swing was permitted to assess coordination improvements. These tests were selected as they evaluate lower limb power and coordination, fundamental components for soccer performance, particularly in jumping for headers and explosive movements [23]. Slinde and colleagues [24] showed a high test–retest stability coefficient (range 0.80–0.98) for CMJ performances.
- 15-Meter Linear Sprint (LS) and 15-Meter Sprint with Ball Control (LSB). For the sprint test, the players completed a 15 m linear sprint in the shortest possible time. These tests measure fundamental speed capabilities and technical-coordinative abilities essential for soccer performance, with the ball control variant specifically assessing the integration of technical skills under speed demands [25]. The time spent in the sprint test was detected by a photocell system (Witty, Microgate, Bolzano, Italy). The fastest time among multiple trials was recorded for analysis. Altmann and colleagues [26] showed a high test–retest stability for these tests.
- 15-Meter Agility Test (AT) and 15-Meter Agility Test with Ball Control (ATB). Agility was assessed using a 15 m slalom sprint. These tests evaluate the ability to change direction rapidly, a crucial skill in soccer for evading opponents and creating space, with the ball variant assessing technical execution under coordinative demands [25]. The time spent in the sprint test was detected by a photocell system (Witty, Microgate, Bolzano, Italy). The fastest time among multiple trials was recorded for analysis. Altmann and colleagues [26] showed a high test–retest stability for these tests.
- Sit and Reach Test (SeR). Lower limb and spine flexibility were evaluated using the Sit and Reach test. Players sat with their legs extended and their feet resting on a graduated panel, and their best maximum forward reach distance among multiple attempts was measured [27]. This test was included as flexibility is important for injury prevention and overall movement quality in young athletes.

### 2.3. Statistical Analysis

Variables were not normally distributed, even after natural log transformation. Thus, a non-parametric Kruskal–Wallis test for independent samples was used to detect differences among age groups. Considering the total number of five groups and the smallest group sample size, a post hoc power analysis conducted with G*Power (v 3.1.9.7) indicated a statistical power of 0.93 for detecting a large effect size (f = 0.40), with an alpha error probability of 0.05.

Since significant differences between younger and older athletes could be expected due to athletes’ growth, the post hoc analysis by the non-parametric Mann–Whitney U test was employed to determine whether differences existed only between adjacent age categories (U9 vs. U10, U10 vs. U11, U11 vs. U12, U12 vs. U13). To investigate differences within the same age category, the Kruskal–Wallis test for independent samples was used to assess differences among all subgroups. For parameters showing significant differences, post hoc comparisons between subgroups (Y vs. O) within each age category (e.g., U9Y vs. U9O, U10Y vs. U10O, etc.) were conducted using the Mann–Whitney U test. Statistical significance was set at *p* ≤ 0.05. The same statistical procedure was then applied to detect significant differences between the subgroups classified according to birth quarter.

## 3. Results

### 3.1. Differences Between Age Groups

**Figure 1:**
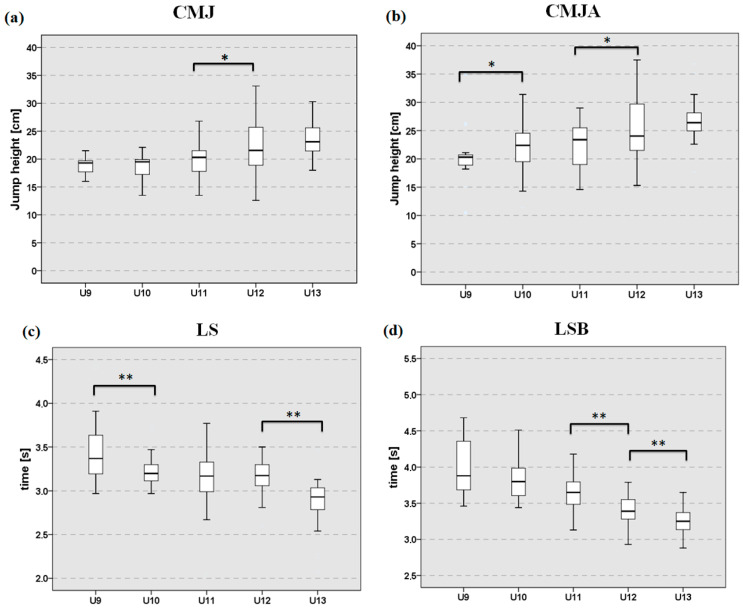

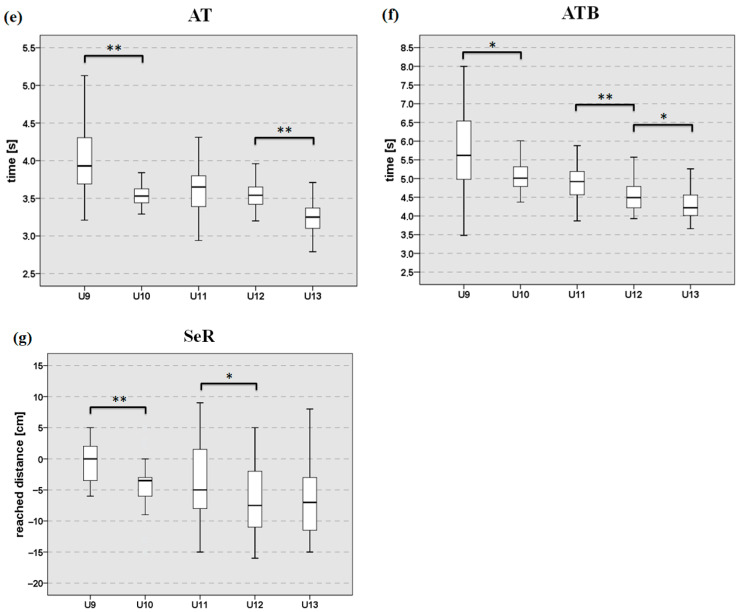
Median values and interquartile range of the five age groups. (**a**) CMJ with blocked arms. (**b**) CMJ with arm swing; (**c**) 15 m linear sprint without ball; (**d**) 15 m linear sprint with ball; (**e**) Agility test without ball; (**f**) Agility test with ball; (**g**) Sit and Reach test. Significant differences between adjacent age groups: * *p* ≤ 0.05; ** *p* ≤ 0.01.

Kruskal–Wallis test showed significant differences among groups in all measured variables (*p* < 0.01); thus, the Mann–Whitney post hoc test between categories of adjacent ages was performed for each parameter. The *p*-values and their effect size [28] are reported for significant results. For CMJ, a significantly greater jump height in U12 was observed compared to U11, with a small-to-medium effect size (*p* = 0.032, η^2^ = 0.052) (Figure 1a). In the CMJA, a significantly better performance of medium effect size was observed in U10 compared to U9 (*p* = 0.045, η^2^ = 0.074), and in U12 compared to U11 (*p* = 0.022, η^2^ = 0.059), suggesting that age accounts for a moderate proportion of the variability in CMJA for these groups (Figure 1b). In the 15m LS test, U10 showed significantly faster sprint times than U9 (*p* = 0.01, η^2^ = 0.124) and U13 outperformed U12 (*p* < 0.01, η^2^ = 0.388), indicating a medium-to-large effect size (Figure 1c). In the 15m LSB test, significant differences in medium-to-large effect size were observed in the oldest groups, with faster sprint times in U12 compared to U11 (*p* < 0.01, η^2^ = 0.199) and in U13 compared to U12 (*p* = 0.01, η^2^ = 0.087) (Figure 1d). In the AT, significantly better performance was observed in U10 compared to U9 (*p* < 0.01, η^2^ = 0.199) and in U13 compared to U12 (*p* < 0.01, η^2^ = 0.284); both effects were large, indicating that group differences were substantial (Figure 1e). In the ATB, a significantly faster time of medium-to-large effect size was found in U10 compared to U9 (*p* = 0.01, η^2^ = 0.12), in U12 compared to U11 (*p* < 0.01, η^2^ = 0.133) and in U13 compared to U12 (*p* = 0.016, η^2^ = 0.075); indicating that the group differences were meaningful (Figure 1f). Finally, in the SeR test, a significantly greater flexibility was observed in U9 compared to U10 (*p* < 0.01, η^2^ = 0.126), and in U11 compared to U12 (*p* = 0.049, η^2^ = 0.043), indicating a medium effect for the first comparison and a small effect for the second one (Figure 1g). The median values and interquartile ranges of all groups are shown in Table 1.

### 3.2. Differences Within Age Subgroups

The Kruskal–Wallis test showed significant differences among subgroups in all measured variables (*p* ≤ 0.01); thus, for each parameter, a Mann–Whitney post hoc test was performed between subgroups of the same age group.

**Figure 2:**
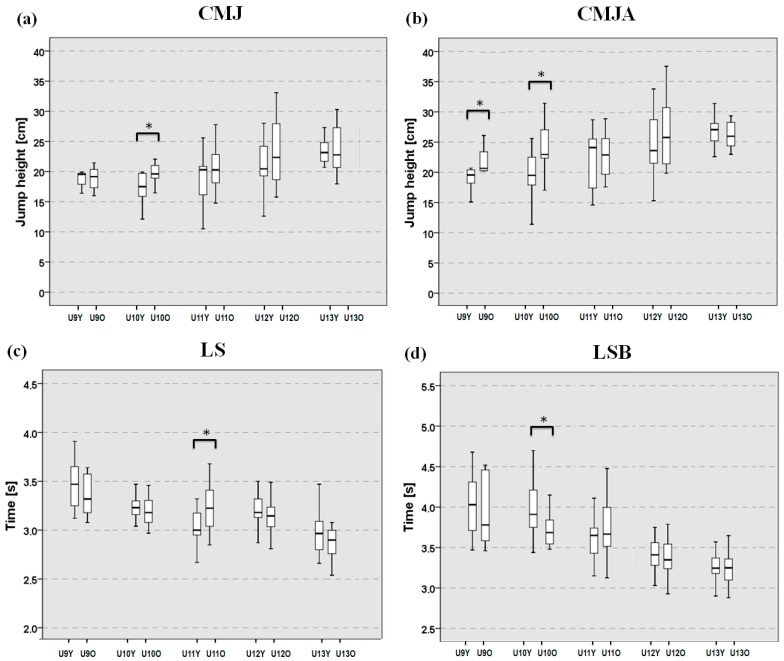

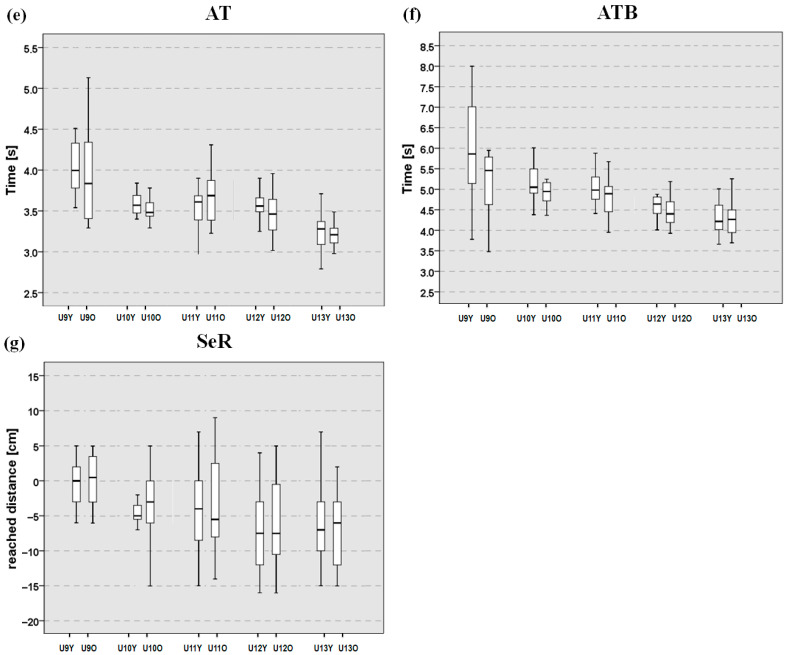
Median values and interquartile range of the ten age subgroups. (**a**) CMJ with blocked arms. (**b**) CMJ with arm swing; (**c**) 15 m linear sprint without ball; (**d**) 15 m linear sprint with ball; (**e**) Agility test without ball; (**f**) Agility test with ball; (**g**) Sit and Reach test. Significant differences between adjacent age groups: * *p* ≤ 0.05.

In the youngest age group (U9), significant differences were observed in the CMJA test (Figure 2b), with subgroup U9O showing significantly higher scores compared to U9Y (*p* = 0.040, η^2^ = 0.18), while no differences were found in the other tests. Among all age groups, U10 athletes showed the greatest number of differences between the two age subgroups. Specifically, subgroup U10O showed significantly better performance in both the CMJ (Figure 2a) (*p* = 0.019, η^2^ = 0.175) and CMJA tests (Figure 2b) (*p* = 0.033, η^2^ = 0.144) compared to U10Y. Moreover, U10O athletes also showed significantly better sprint times in the LSB (Figure 2d) test than those in U10Y (*p* = 0.033, η^2^ = 0.144). In the U11 group, differences between subgroups were found only in the LS test (Figure 2c), with athletes in subgroup U11Y achieving better sprint times than those in subgroup U11O (*p* = 0.016, η^2^ = 0.147). Overall, the effect sizes observed across these significant comparisons were consistently large, reflecting meaningful differences between subgroups. In the oldest age groups (U12 and U13), no significant differences were found between subgroups for any of the assessed parameters. In general, no significant differences between subgroups were detected in AT (Figure 2e), in ATB (Figure 2f), and in the SeR test (Figure 2g). The median values and interquartile ranges of all subgroups are shown in Table 2.

No significant differences were detected from the explorative analyses based on birth quarter, except for the LSB (U12-born Q4 (*n* = 12) vs. U11-born Q1 (*n* = 14): 3.41 ± 0.33 vs. 3.78 ± 0.38; *p* = 0.006, η^2^ = 0.27), indicating a very large effect size. However, given the limited sample size, these results should be treated with caution.

## 4. Discussion

The findings of this research indicate age-related differences in motor tests with medium to large between-group effects for all adjacent ages for speed and agility, while improvements in ball-involving tasks appear only in later ages. These outcomes are consistent with other studies in the literature, demonstrating that some performance markers follow a relatively predictable pattern of improvement, yet are modulated by factors such as motor control, training adaptation, and biological maturation [3,6]. Indeed, for linear sprint and agility tests without the ball, adjacent age-group comparisons showed medium-to-large effects, whereas the ball-involving tasks version shifted these effects only toward older groups (U10–U11, U11–U12). On the other hand, jump performance generally improved with a smaller magnitude of effects. These, taken together, support the non-linear pattern in early adolescence consistent with other longitudinal studies accounting for accelerations and plateaus regarding pre-adolescence [3,6,29,30]. Semester subgrouping analyses revealed meaningful differences primarily in the younger categories (e.g., from 9 to 11 years old, differences in several tests have a large magnitude with η^2^ comprised between 0.14 and 0.18), while no differences were observed in U12-U13. On the other hand, trimester analysis was explorative to verify the magnitude of changes. Indeed, the absence of consistency in the analysis suggest the non-linear trajectory changes that are more evident at the semester level. However, due to the sample size limitation and the contextual factors possible interference with the skill development path, trimester results should be interpreted cautiously [29].

In the considered sample, the within-age pattern is consistent with the over-representation of earlier-born athletes reported in youth soccer [20,21]. That is consistent with the literature, where a six-month age gap is associated with detectable differences only for non-complex explosive tasks among the youngest categories. However, due to subgroup sizes being modest and effects not being uniform, these differences should be interpreted cautiously. Moreover, the yearly semester division comparisons showed that the older categories outperformed the younger, in accordance with the Italian elite benchmarks [20]. Indeed, in Italy, RAEs are pronounced in top youth categories, tending to attenuate toward Serie A, which is consistent with our pattern [20,21]. This picture supports the interpretation that in younger groups, maturational advantages could induce early selection bias that then will cushion with age [21]. Despite that, differences were measure-specific and not consistent across all outcomes. Indeed, beyond maturational timing, semester-based differences might reflect selection and opportunity mechanisms that advantage relatively older players within the cut-off system. Accordingly, soccer evidence shows an over-representation of early born athletes, with magnitudes peaking during adolescence, being consistent with greater exposure, competitive minutes and perceived competence, reinforcing development over time [31,32]. On the other hand, within U11, the younger subgroup was faster in the linear sprint test than the older counterpart. This pattern could be interpreted in light of the non-linear variation typical of early adolescence that is characterized by spurts and plateau in terms of physical skills maturation, especially in easier tasks [6,29]. Accordingly, this difference might reflect the contextual influences that can modulate performance appearance and RAE prevalence [33].

To account for this mismatch between age groups, several authors propose other grouping strategies, challenging conventional cutoffs. For instance, Lovell [15] proposed using markers of physical maturity (height, weight, and body composition), while Perroni [34] and Malina [35] emphasize skeletal or pubertal assessments to refine groupings. Others, such as Malina [36], suggest classification based on skill level, and Sierra-Díaz with colleagues [32] propose introducing a quota system for later-born players to ensure equal opportunities. These approaches may help clubs promote talent discovery and tailor training to individual skills development. Nevertheless, power and speed development, including object control (e.g., dribbling), appear to benefit from significant differences between the ages of 10 and 12. These findings are consistent with models hypothesizing that strength and endurance often precede the emergence of fine coordination skills [29,30]. With adequate muscle capacity, players may be better equipped to adapt to drills demanding complex footwork, rapid decision-making, and sensorimotor integration [37].

Together, the results reported here should help to contextualize the group considered: large quarter/semester gaps in pre-adolescence are typical in competitive pathways but should be managed to avoid talent loss among relatively younger players. Potentially, in daily practical work, athletes’ selection could be influenced more by multiple checkpoints and development trajectories rather than absolute values, given the athlete-specific skill improvements during adolescence [3,6]. Indeed, given that senior-level RAE focuses during career beginning, reducing early selection pressure in preadolescence may help contextualize evaluation in similar settings.

### Limitations

Subgrouping strategy (i.e., half-year cohorts and birth quarters) reduced the size of each subgroup, which may have masked additional differences. Moreover, the cross-sectional design provides only a snapshot of each athlete’s development, limiting the possibility to identify individualized trajectories over time. The lack of maturation markers (e.g., height, weight, skeletal age, etc.) has to be addressed as a barrier for generalizability. This study did not assess passing, shooting, or tactical decision-making, with ball-involving tests being considered as dribbling-related tests appropriate for 8 to 12-year-old groups. Finally, findings must be interpreted in light of the single-club male-only, cross-sectional design.

## 5. Conclusions

This study shows a progressive, though not strictly linear, path of motor development in youth soccer. Indeed, despite motor performance improving between each consecutive age group, the gain magnitude was not linear (e.g., ball-involving tasks tend to consolidate later) [3]. Semester grouping revealed meaningful differences in younger categories, while trimester contrasts were mainly trivial. When involving the ball, skills tend to consolidate later than easier speed and power. Therefore, potential practical implications should be modest and context-specific for clubs and academies.

### Future Directions

To clearly map growth-related changes, a longitudinal design with larger samples should be adopted to track within-player paths and quantify how motor performance and the RAE evolve over time. It would also be valuable to account for the role of external factors such as training consistency, injury history, training disruptions when interpreting these outcomes [33]. Finally, future studies should include validated technical skills (e.g., passing, shooting) and game-based measures (e.g., decision-making tools) to delineate object involvement in these fields. With this strategy accounting for longer times and greater samples, a better understanding of motor-skill progression will be achieved.

## Figures and Tables

**Table 1:** Median and interquartile range values of age groups.

Groups	CMJ [cm]	CMJA [cm]	LS [s]	LSB [s]	AT [s]	ATB [s]	SeR [cm]
U9	19.3 [17.5–19.7]	20.3 [18.7–20.7] *	3.37 [3.16–3.64] *	3.88 [3.66–4.4]	3.93 [3.66–4.33] *	5.62 [4.82–6.85] *	0 [−4–+2] *
U10	19.5 [17.1–19.9]	22.4 [19.5–24.6]	3.2 [3.11–3.31]	3.8 [3.59–3.99]	3.53 [3.44–3.64]	5.01 [4.75–5.38]	−3 [−6–−2]
U11	20.3 [17.5–21.6] *	23.4 [18.7–25.6] *	3.2 [3.0–3.3]	3.65 [3.48–3.8] *	3.65 [3.38–3.86]	4.92 [4.52–5.25] *	−5 [−8–+2] *
U12	21.6 [18.8–26.0]	24.1 [21.4–29.8]	3.18 [3.06–3.3] *	3.39 [3.28–3.55] *	3.54 [3.4–3.65] *	4.49 [4.21–4.79] *	−7.5 [−11.3–−2]
U13	23.1 [21.4–25.7]	26.4 [24.7–28.2]	2.93 [2.77–3.04]	3.25 [3.1–3.37]	3.25 [3.09–3.37]	4.22 [4.0–4.61]	−7 [−12–−3]

CMJ = Countermovement Jump; CMJA = Countermovement Jump with arm swing; LS = Linear Sprint; LSB = Linear Sprint with ball; AT = Agility test; ATB = Agility test with ball; SeR = Sit and Reach. * *p* ≤ 0.05 compared to the subsequent older age category.

**Table 2:** Median and interquartile range values of age subgroups.

Groups	Subgroups	CMJ [cm]	CMJA [cm]	LS [s]	LSB [s]	AT [s]	ATB [s]	SeR [cm]
U9	U9Y (*n* = 14)	19.6 [17.5–19.7]	19.9 [18.2–20.4]	3.39 [3.16–3.65]	4.02 [3.71–4.31]	3.93 [3.72–4.33]	5.86 [5.14–7.01]	0.0 [−4.0–+2.0]
	U9O (*n* = 9)	19.2 [17.2–21.0]	20.75 [20.3–24.7] *	3.32 [3.16–3.61]	3.78 [3.55–4.49]	3.84 [3.40–4.43]	5.46 [4.58–5.87]	0.5 [−3.5–+3.8]
U10	U10Y (*n* = 15)	17.5 [15.6–19.8]	19.5 [17.5–22.6]	3.23 [3.15–3.31]	3.91 [3.70–4.24]	3.57 [3.46–3.71]	5.05 [4.83–5.56]	−5 [−6.0–−3.0]
	U10O (*n* = 16)	19.7 [18.8–21.1] *	22.9 [22.3–28.0] *	3.18 [3.07–3.31]	3.69 [3.54–3.86] *	3.48 [3.43–3.60]	4.95 [4.72–5.18]	−3 [−7.5–0.0]
U11	U11Y (*n* = 15)	20.3 [16.1–21.0]	24.1 [17.3–25.6]	3.0 [2.92–3.18]	3.65 [3.38–3.74]	3.61 [3.28–3.70]	4.98 [4.72–5.31]	−4 [−9.0–0.0]
	U11O (*n* = 24)	20.3 [18.1–22.9]	23.0 [19.7–26.2]	3.23 [3.04–3.42] *	3.67 [3.51–4.01]	3.69 [3.39–3.88]	4.9 [4.46–5.10]	−5.5 [−8.0–+2.8]
U12	U12Y (*n* = 22)	20.5 [19.0–24.3]	23.6 [21.3–28.8]	3.18 [3.12–3.34]	3.41 [3.28–3.57]	3.56 [3.48–3.66]	4.64 [4.40–4.83]	−7.5 [−12.8–−2.8]
	U12O (*n* = 20)	22.4 [18.6–28.0]	25.7 [21.2–31.0]	3.15 [3.03–3.24]	3.35 [3.22–3.55]	3.465 [3.27–3.65]	4.405 [4.19–4.75]	−7.5 [−12.8–−0.3]
U13	U13Y (*n* = 22)	23.2 [21.7–25.0]	27.1 [25.1–28.2]	2.97 [2.79–3.09]	3.25 [3.16–3.39]	3.28 [3.08–3.37]	4.215 [4.02–4.62]	−7 [−10.3–−2.3]
	U13O (*n* = 13)	22.8 [20.5–22.8]	25.9 [24.2–28.8]	2.9 [2.74–3.0]	3.25 [3.08–3.39]	3.21 [3.06–3.36]	4.27 [3.95–4.72]	−6 [−13.0–−3.0]

CMJ = Countermovement Jump; CMJA = Countermovement Jump with arm swing; LS = Linear Sprint; LSB = Linear Sprint with ball; AT = Agility test; ATB = Agility test with ball; SeR = Sit and Reach. Subgroup Y is younger than subgroup O. * *p* ≤ 0.05 compared to the corresponding subgroup of the same age category.

## Data Availability

Data are available under request to the corresponding author. To preserve the integrity of the ongoing work, the dataset cannot be openly shared at this stage.
